# Environmental and Ontogenetic Effects on Intraspecific Trait Variation of a Macrophyte Species across Five Ecological Scales

**DOI:** 10.1371/journal.pone.0062794

**Published:** 2013-04-23

**Authors:** Hui Fu, Guixiang Yuan, Jiayou Zhong, Te Cao, Leyi Ni, Ping Xie

**Affiliations:** 1 Donghu Experimental Station of Lake Ecosystems, State Key Laboratory of Freshwater Ecology and Biotechnology, Institute of Hydrobiology, The Chinese Academy of Sciences, Wuhan, China; 2 Jiangxi Institute of Water Sciences, Nanchang, China; 3 Graduate School of Chinese Academy of Sciences, Beijing, China; Brigham Young University, United States of America

## Abstract

Although functional trait variability is increasingly used in community ecology, the scale- and size-dependent aspects of trait variation are usually disregarded. Here we quantified the spatial structure of shoot height, branch length, root/shoot ratio and leaf number in a macrophyte species *Potamogeton maackianus*, and then disentangled the environmental and ontogenetic effects on these traits. Using a hierarchical nested design, we measured the four traits from 681 individuals across five ecological scales: lake, transect, depth stratus, quadrat and individual. A notable high trait variation (coefficient variation: 48–112%) was observed within species. These traits differed in the spatial structure, depending on environmental factors of different scales. Shoot height and branch length were most responsive to lake, transect and depth stratus scales, while root/shoot ratio and leaf number to quadrat and individual scales. The trait variations caused by environment are nearly three times higher than that caused by ontogeny, with ontogenetic variance ranging from 21% (leaf number) to 33% (branch length) of total variance. Remarkably, these traits showed non-negligible ontogenetic variation (0–60%) in each ecological scale, and significant shifts in allometric trajectories at lake and depth stratus scales. Our results highlight that environmental filtering processes can sort individuals within species with traits values adaptive to environmental changes and ontogenetic variation of functional traits was non-negligible across the five ecological scales.

## Introduction

Trait-based approaches have been widely applied to investigate how community structures and population dynamics change along environment gradients [1], [2]. Keddy [3] has suggested that plant species can distribute in specific environment conditions, largely attributing to their inherent range of functional traits. This view has been supported by a number of evidences that plant communities generally show trait-based patterns distribution along environmental gradients [4], [5], [6], [7]. These functional traits are variable between and within species. Numerous studies have focused on the interspecific trait variation to determine the importance of different trade-offs in structuring community dynamics [6], [8], [9], [10], [11]. However, there is growing evidence that intraspecific trait variation is non-negligible [12], [13], [14] and even important in many aspects of ecological processes, such as population dynamic [15], [16], [17], [18], species coexistence [19], [20], [21], functional diversity [22], [23] and ecosystem function [24], [25], [26].

Many ecological patterns are generally scale-dependent [27], [28], and the habitats of plant species can span across a broad range of ecological scales, with distinct environment gradients, indicating that the amount of trait variation would differ among different scales. Messier *et al* [12] has systematically clarified the potential scale-dependent aspects of trait variation across six hierarchical ecological scales (leaf, strata, tree, species, plot, site), while Albert *et al* [14] has quantified the extent, spatial structure and source of intraspecific trait variation across three ecological scales (individual, subpopulation, population). Both studies show a large variability of functional traits within species, and the performance of individuals and populations is largely dependent on the environmental gradients under specific ecological scale. Identifying which scales account for the most variation in functional traits will help check the assumptions as predicted by many existing theories [1], [12], [29], [30], [31]. For example, under the environmental filtering paradigm, filtering processes sort species with traits values close to optimal trait values [4], [6], [7]. More recently, Violle *et al* [32] advocate moving beyond species-based to individual-based community ecology within a new spatially explicit framework, which models community assembly via two different filters: external (filtering processes outside the community) and internal (filtering processes inside the community). According to this framework, filtering processes operate on individuals instead of species, implying that ecological filters can sort individuals within species. Therefore, the overall distribution of intraspecific trait variation across ecological scales might be established by both external and internal filtering processes, while the relative amount of intraspecific trait variation largely depend on the environmental gradients in specific ecological scale.

Besides the characters of scale-dependent, functional trait variation is dramatically size-dependent [33], [34], [35], [36] and change over the course of growth and development (ontogenetic drift) [37]. Numerous studies have suggested that only when we account for the effects of plant size, the adjustments of functional traits would be adaptive responses to environment gradients such as light, nutrient and water [33], [38], [39]. It can thus be assumed that ontogenetic variation should be an important source of intraspecific trait variation [33]. However, ontogenetic variation has been usually disregarded in the responses of functional traits to environmental factors [7], [12]. Due to the differences in the climate and environmental gradients in different ecological scales, plant vary greatly in the development stage and rate both between and within species, which may account for the distinct ontogenetic variability of functional traits among ecological scales. Although the responses of functional traits to environmental gradients are potentially constrained by plant development or ontogeny, disentangling the effects of environment and ontogeny on the functional trait variation has yet to be fully investigated.

*Potamogeton maackianus* A. Been (Potamogetonaceae) is a clonal and perennial submersed macrophyte widely distributed in East Asia [40]. It is one of the dominate species and mostly forms mono-specific mats in many shallow lakes in the middle reaches of Yangtze River and some plateau lakes in Yunnan Province, China [41]. The habitats of this species span across a broad range of ecological scale, involving different environmental gradients (i.e., nutrients, water depth, latitude and altitude). Furthermore, this species shows a highly phenotypic plasticity and comparable high genetic diversity as adaptive responses to freshwater habitats [41], [42], [43], [44], [45]. This can therefore be considered a special case: 1) where there is an important environmental variability but no species turnover, because phylogenetic inertia is absent and only one macrophyte species persists in the studied areas; 2) where it is more effective to determine the ontogenetic variability of traits within a single species, because the interspecific differences in ontogenetic trajectories of traits may confound results of trait-environment relationships [13], [18].

In this study, we aimed to quantify the scale- and size-dependent aspects of intraspecific variation of functional traits such as shoot height, branch length, leaf number and root/shoot ratio in *P. maackianus* across five nested ecological scales (lake, transect, depth stratus, quadrat, individual). We applied a general linear mixed model to the trait variance to disentangle the environmental and ontogenetic effects on these traits in each scale, by considering or not considering plant size as the covariate. Subsequently, we analyzed the significance of environmental and ontogenetic effects on these traits by linking the shifts of allometric trajectories between plant size and traits across ecological scales. First we ask, how is intraspecific variability of measured traits structured spatially across the five ecological scales, and which traits are the most responsive ones in different scales? Second, how much of intraspecific trait variability is accounted for by environment and ontogeny respectively across the five ecological scales, and is ontogenetic variance negligible? Third, how are the allometric trajectories of traits varied across the five ecological scales? We hypothesized that (1) environmental filtering processes could sort individuals with traits values close to optimal trait values within species (e.g., water depth gradients sort individuals with longer shoot in deeper water, and shorter shoot in shallower water); and (2) ontogenetic variation of functional traits was non-negligible across the five ecological scales.

## Materials and Methods

Our study complies with the current laws of China and with international rules. No specific permissions were required for the described field studies; all samples were obtained from publicly accessible waters and the studied species is not endangered or protected.

### Study location

Present study was carried out in Erhai Lake (25°52′N, 100°06′E) in Yunnan Province and Niushanhu Lake (30°20′N, 114°30′E) in Hubei Province, China. In Erhai Lake, submersed macrophytes had experienced dramatic changes in the distribution and species composition during the last 50 years, due to artificial regulation of water levels and anthropogenic eutrophication [46], [47]. In 1950–60s when the lake was turbid, oligotrophic and at high water levels, macrophyte communities were limited to the shallow water (about 3 m depth) and dominated by *Potamogeton pectinatus* Linn., *Najas marina* Linn. and *Ottelia acuminata* (Lévl. et Vant.) Dandy [48]. After a dry period during 1978–1981, many other macrophyte species inhabited successfully in this lake and expanded rapidly to cover more than 60% area of the lake [46], [48]. In 1980s when the lake was clear and at low water levels, *Vallisneria natans*, *Hydrilla verticillatta* and *P. maackianus* became dominant and expanded to cover more than 30% area (75 km^2^) of the lake, distributing to water depth of 8 m [47], [49]. In the last decade, the lake started to become turbid (early eutrophication stage) and the water level increased by 2 m, submersed vegetation reduced by 80% in area and distributed to water depth of no more than 5 m, with *P. maackianus* being the predominant species.

Niushanhu Lake, a subtropical shallow lake in the middle reaches of Yangtze River, China, has been an large arm located at north of Liangzi Lake (30°3′–30°19′N, 114°26′–114°38′E) [50]. In the 1960s, almost the whole lake bottom was covered by submersed macrophytes, mainly *H. verticillatta*, *V. natans*, *N. marina* and *Najas minor* [51]. Since 1979, however, this lake has been separated artificially from Liangzi Lake by a dam. During the 1980s, the predominant species was replaced by *P. maackianus*, with *Myriophyllum spicatum* L. and *V. natans* being the companion species [50], [51]. In the following years, *P. maackianus* was always the most dominant species. During the study period, large beds of *P. maackianus* population covered almost the total bottom surface and extended from 0.2 to 3.5 m depth range in this lake.

The average values and ranges of selected morphometrical and limnological characteristics in Erhai Lake and Niushanhu Lake during the study period were showed in Table 1. The macrophyte communities of these two lakes were sampled once during the summer (June to September) of either 2008 (Niushanhu Lake) or 2009 (Erhai Lake). Using existing bathymetric maps, we randomly located 24 and 224 sampling plots (25 m^2^) in Niushanhu Lake and Erhai Lake, respectively. The total macrophyte coverage was calculated by planimetry of the area above the mean depth of maximum colonization. To estimate the coverage of *P. maackianus*, we divided the sum of the frequency occurrence of this species by the sum of the frequency occurrence of macrophytes at those plots where it grows, and then multiplied the total macrophyte coverage. The biomass of *P. maackianus* within a 0.2 m^2^ quadrat was collected at each plot. Sampled plants were spun to remove excess water and weighted to the nearest 0.10 kg fresh weight (FW). The biomass of total macropytes was calculated as the average macorphytes biomass of all plots multiplied by the total macrophyte coverage. In each plot, the physical parameters in water (water depth, water temperature, dissolved oxygen, Secchi depth and pH) were assessed immediately *in situ* [52]. For all plots, water samples were transported to laboratory and assayed. These samples were analyzed for total algal biomass, total nitrogen content (TN), total phosphorus content (TP), ammonium nitrogen content (NH_4_-N) in water and chemical oxygen demand of water (COD) [52]. Total algal biomass was measured as the chlorophyll *a* concentrations [52]. TP in the lake water was measured by colorimetry after digestion of the total samples with K_2_S_2_O_8_+NaOH to orthophosphate. TN was digested simultaneously with TP. After digestion, TN was measured as nitrate and absorbance was measured at 220 nm [52]. For the analysis of NH_4_-N, water samples were filtered through a Whatman (Middlesex, UK) GF/C grass fiber membrane (0.45-µm pore diameter). NH_4_-N was analyzed by the Nessler method [52].

**Table 1:** The average values and ranges of selected morphometrical and limnological characteristics in Erhai Lake and Niushanhu Lake during the study period.

Parameter	Erhai Lake		Niushanhu Lake	
	Average	Range	Average	Range
Altitude	1973		27	
Surface area (km^2^)	249.76		38	
Storage capability (m^3^)	25.4×10^8^		1.18×10^8^	
Growth days (day)	320		210	
Coverage of *P.maackainaus*	2.95%		26.50%	
Total macrophytes coverage	5.20%		39.50%	
Biomass of *P.maackainaus* (g FW m^−2^)	12827	1250–25416	8650	6283–11183
Biomass of total macrophytes (kg FW)	2.22×10^8^	1.52×10^8^ −3.04×10^8^	0.34×10^8^	0.25×10^8^ −0.44×10^8^
Water depth (m)	10.8	0–21.5	3.5	0–4.5
Temperature (**°C**)	18.74	8.9–27.3	21.76	2.15–32.85
Dissolved oxygen (mg L^−1^)	6.8	5.05–9.12	8.79	8.1–10.5
Secchi depth (cm)	1.45	1.15–2.85	2.5	1.5–3.6
pH	8.6	8.1–9.7	8.1	7.3–8.6
Total algal biomass (mg L^−1^)	2.84	0.31–4.36	0.201	0.022–0.35
TN (mg L^−1^)	0.52	0.20–0.96	0.36	0.22–0.67
TP (mg L^−1^)	0.027	0.017–0.047	0.008	0.001–0.019
NH_4_-N (mg L^−1^)	0.14	0.09–0.20	0.08	0.01–0.12
COD (mg L^−1^)	3.6	2.35–4.86	1.6	0.6–2.2

Growth days indicate the number of days with the average daily water temperature of more than 10**°C**; FW: fresh weight; TN: total nitrogen content in water; TP: total phosphate in water; NH_4_-N: ammonium nitrogen content in water; COD: chemical oxygen demand of water.

### Ecological scales and traits selection

We assessed variation in shoot height, branch length, root/shoot ratio and leaf number across five hierarchical ecological scales: 1) among individuals (ramets) within a quadrat; 2) among quadrats within a depth stratus; 3) among depth strata within a transect; 4) among transects within a lake; 5) between lakes. A genet/clone (for many clonal macrophyte species) may be very large and cover the whole shallow water (e.g., from 0.1 to 4.0 m depth), at least for this species. In addition, a genet/clone might stand for many years in a lake [42] and the historical environmental changes may confound the trait variations across ecological scales. Furthermore, this species showed a remarkably high population density at less than 4 m depth, with 5000–12000 shoots m^−2^ in Erhai Lake as our field observations, resulting in the difficulty to disentangle the one genet from others under water. Therefore, it is of great difficulty to nest genet/clone scale to any other scales, and the quadrat and individual (ramet in this study) scales were sampled in our study. These five scales contain an array of spatial factors both with explicit environmental gradients (depth strata and lakes) and with no immediately obvious environmental gradient (individual, quadrat and transect). Transects within our two lakes were established systematically at different arms, staying within the same habitat and subject only to local topographic variation, whereas the two lakes themselves were purposely arrayed on distinct primary productivity. Depth strata within each transect were characterized by water depth gradients, while quadrats within each depth stratus by micro-habitat heterogeneity. This nested design contained a logically nested spatial structure, which can be decomposed into two operational filters: the external (lake, transect and depth stratus) and internal (quadrat and individual) filters. The external filter sorts population mean values of traits from regional pool, while the internal filter includes all local processes within population, such as micro-habitat heterogeneity and density-dependent processes [32]. These scales are the most commonly studied by ecologists and the four traits were chosen to represent major spectrums of plant strategies [8], [11], [53], [54], [55]. Both shoot morphology (shoot height, branch length and leaf number) and biomass allocation (root/shoot ratio) are key traits in the life history strategy and have the advantage of well-established sampling protocols with low error variance [56]. Shoot height, branch length and leaf number are pivotal in the canopy formation and closely correlated to competition, light interception and photosynthetic area [8], [57], [58], [59]. Branch length and leaf number can also be closely associated with meristem allocation to branch and leaf tissue, and their variability may mirror the plasticity of meristem fate or developmental program across environmental gradients [60]. Root/shoot ratio reflects the fundamental tradeoff in investing resources in aboveground vs. belowground tissue and therefore has been argued to be the root variable governing correlations among the traits in the life history strategy [39].

### Field sampling and traits determination

To capture trait variation between lakes, we sampled two lakes located at south China, Erhai Lake and Niushanhu Lake (Table 1), which was mainly characterized by their distinct productivity. Erhai Lake had higher productivity, with longer growth period, higher nutrients and plant abundance (macrophyte and phytoplankton) and lower water transparency, relative to Niushanhu Lake (Table 1). We chose the lakes to assess the relative importance of productivity on trait variability; nutrition, water transparency and phytoplankton are productivity measures known to have a large influence on phenotypic traits [41], [44], [55], [58], [61], [62]. To assess the variation among transects within a lake, we sampled three transects in Erhai Lake and four in Niushanhu Lake. To measure variation among depth strata within a transect, at each transect we sampled 3–10 25 m^2^ plots located along water depth gradients in each 0.5 m interval as a depth stratus, extending from 0 m to 3.5 m (Niushanhu Lake) or 5.0 m (Erhai Lake) water depth. To capture variation among quadrats (micro-habitat) within a depth stratus and among individuals within a quadrat, we sampled 3–10 individuals from each of three quadrats located randomly in the plots. To control for temporal variation in traits that occurs between seasons, all data were collected during the growth season (July) of 2008 in Niushanhu Lake, and of 2009 in Erhai Lake. Individual functional traits may vary in relation to the age, size or developmental stage of a plant, which is another important source of variation [60] and can be denoted as ontogenetic variance. In this study, we assessed the ontogenetic variance as the functional trait variations related to plant size. For this point, we collected quasi-randomly the individuals from all of samples and weighted the measured traits by plant size to assess the ontogenetic variance. However, it was unavoidable that we would artificially favor to sample the fine individuals. Therefore, the total trait variances, especially the variations among individuals within quadrat, might be underestimated in this study. Samples were collected using a rotatable reaping hook (diameter  = 0.5 m, area  = 0.2 m^2^), and it can usually uproot the vast majority of individuals within the quadrat in mud. We sorted carefully 3–10 intact and healthy individuals as much as possible from the uprooted plants of each quadrat. We sampled a total of 2 lakes, 7 transects, 47 depth strata, 141 quadrats and 681 individuals (see Table 2 for additional information on sample size).

**Table 2:** The range of latitude and longitude, area (km^2^), number of depth stratus (N) and depth range (m) within each depth stratus in each transect of two lakes: e1, e2, e3 in Erhai Lake and n1, n2, n3, n4 in Niushanhu Lake.

Transect	North latitude	East longitude		Area (km^2^)	Depth stratus (N)	Depth range (m)
Erhai Lake						
e1	25°55′30.82″ −25°56′33.0″		100° 6′36.95″ −100° 7′31.62″	1.8	8	0.5–4
e2	25°55′54.58″ −25°57′63.20″		100° 8′20.8″ −100° 9′44.91″	2.1	10	0.5–5
e3	25°36′24.6″ −25°37′38.75″		100°13′57.8″ −100°14′04.0″	1.5	10	0.5–5
Niushanhu Lake						
n1	30°20′8.82″ −30°21′33.0″		114° 28′26.15″ −114° 29′35.12″	1.25	6	0.5–3
n2	30°20′12.1″ −30°21′19.0″		114° 29′45.31″ −114° 30′24.43″	0.85	4	1–3.5
n3	30°21′06.31″ −30°21′58.0″		114° 30′36.56″ −114° 31′15.31″	1.12	5	1–3.5
n4	30°20′56.21″ −30°21′11.30″		114° 32′06.46″ −114° 32′55.37″	0.75	4	0.5–2

For each intact individual (ramet), shoot height (cm) was calculated as the distance from the basal stem to the top of photosynthetic tissues, branch length (cm) as the sum of branch stem lengths and leaf number (N plant^−1^) as the sum of green leaves. We separated each individual into roots and shoots and weighted after oven-dried at 80**°C** for 48 hours. Root/shoot ratio (g g^−1^) was calculated as the ratio of roots and shoots dry weight. Total biomass per plant (here after called plant size) was the sum of roots and shoots dry weight.

### Statistical analysis

Using a restricted maximum likelihood (REML) method in the ‘lme’ function of R (version 2.6.1) and data normalized by log10 transformations, we fitted a general linear mixed model to the variance across five scales nested one into another (i.e. nested ANOVA with random effects) in this increasing order: individual, quadrat, depth stratus, transect and lake. To assess the size-corrected variation of traits in each scale, we fitted an alternate general linear mixed model to the variance across five scales nested one into another in the same order, with plant size as the covariate. This size-corrected variation might include both environmental variation and covariation. Because the covariation can be understanded by the effects of environment on the allometric relationships between plant size and functional traits, we denoted the size-corrected variation as environmental variation in this study. A variance component analysis was performed on this model using the ‘varcomp’ function of R (R Development Core Team 2007). Therefore, we calculated the ontogenetic variance from the equations:

Ontogenetic variance*_ij_*  =  Total variance*_ij_* – Environmental variance*_ij_*,

where *i* and *j* indicates the *i* traits and *j* scale. In general, in a nested ANOVA the variance components represent the variances around the means or sized-corrected means for each level. We also computed coefficient of variation (CV) for each trait as a complementary index to interpret intraspecific variation.

Standardized major axis (SMA) slope-fitting techniques were used for the allometric analyses [63]. Allometric relationships between traits are generally understood as exponential relationships described by the equation y = β x ^α^, or more commonly Ln (y)  =  Ln (β) +α Ln (x) [63], where x (plant size) and y (response variables) are the two traits, α is the scaling coefficient (slope) and β is a regression constant (intercept) [64]. Noting that the change in y with respect to difference in plant size x (i.e. Δy/Δx) equals α β x^α−1^, the magnitude of y will be independent of intra- or interspecific differences in x when α = 1.0; it will increase disproportionately with increasing x when α>1.0 (said increasing return); and it will fail to keep pace with intra- or interspecific increases in x when α<1.0 (said decreasing return). In this study, to test whether there is any significant shifts in the allometric relationships between plant size and the four traits across lakes, transects or depth strata, we used a likelihood ratio (LR) method to determine the differences in the SMA slopes [65]. When there were parallel slopes between treatments within each scale (test for homogeneity, *P*>0.05), differences in intercept were tested by *t* test as demonstrated by Warton et al. [64]. The statistical software (S)MATR was used for all the analyses [66].

## Results

**Figure 1:**
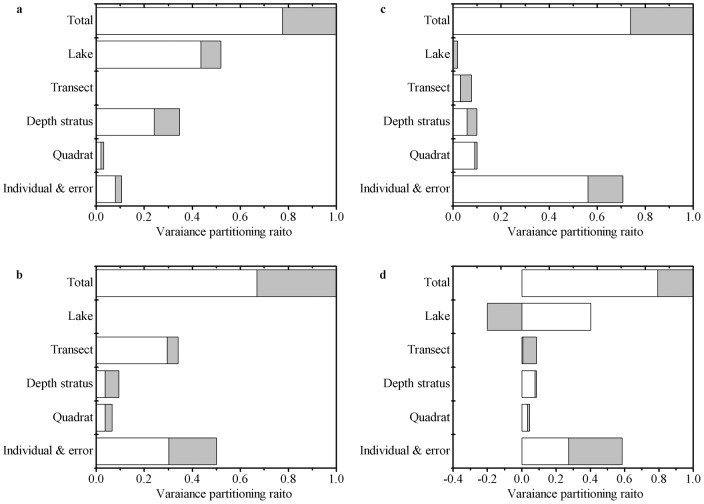
Variance partitioning ratio of individual traits studied across five ecological scales. (a) shoot height; (b) branch length; (c) root/shoot ratio; (d) leaf number. Open part of the columns corresponds to environmental variances, and grey part to ontogenetic variances.

Coefficient variation was 0.63 for plant size, 0.48 for shoot height, 1.21 for branch length, 0.66 for root/shoot ratio and 0.91 for leaf number. The variance partitioning ratios strongly differed depending on the scale and the trait being considered. Lakes account for 52% and 20% respectively of total variance in shoot height (Fig. 1a) and leaf number (Fig. 1d), depth strata for 35% and 10% respectively of total variance in shoot height (Fig. 1a) and root/shoot ratio (Fig. 1c), while transects for 34% of total variance in branch length (Fig. 1b). Note that the percent of total variance at individual scale, representing the variations among individuals within quadrats, ranged from 11% (for shoot height) to 71% (for root/shoot ratio) (Fig. 1a, c).

Decomposition of total variance in traits demonstrates that the trait variance caused by environment is nearly three times higher than that caused by ontogeny, with ontogenetic variance ranging from 21% (for leaf number) to 33% (for branch length) of total variance (Fig. 1b, d). Remarkably, these traits showed non-negligible ontogenetic variations (0–60%) in each ecological scale (Fig. 1). The positive ontogenetic variance indicated a linear relationship between trait and plant size (Fig. 1a–c), while negative ontogenetic variance reflected a nonlinear relationship between trait and plant size (Fig. 1d). In this study, a negative ontogenetic variance was observed in leaf number at lake scale, probably due to the higher trait variation after size corrected than that before size corrected. For example, the relationship between leaf number and plant size may display a unimodel pattern, and plants have very fewer leaflets at both smaller (young plants) and larger (old plants) size.

**Figure 2:**
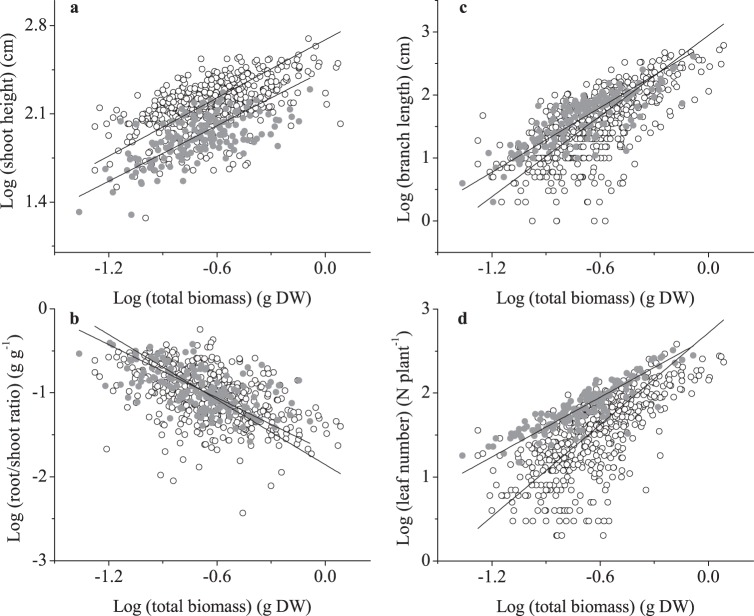
Allometric relationships between traits and total biomass for all individuals in two lakes. (a) shoot height; (b) branch length; (c) root/shoot ratio; (d) leaf number. Open cycle indicates the data in Erhai and closed cycle indicates the data in Niushanhu. Variables were log_10_-transformed prior to model fitting.

At lake scale, there were significant shifts in the allometric trajectories (i.e., slope or intercept) of these traits between lakes (Fig. 2, Table 3). The allometric relationships between shoot height and plant size did no differ between lakes (Fig. 2a, *LR* test  = 0.82, *P* = 0.37), with a common slope of 0.80 [*n* = 677, 95% CI (0.75, 0.85)]. At a given plant size, shoot height was higher at the Erhai than at Niushanhu (*t* test  = 183.7, *P*<0.0001), and significant shift along common slope (*P*<0.0001). Shoot height was also strongly positively dependent on plant size, showing a “decreasing return” with increased plant size (Table 3, α<1, *P*<0.001). Further, the significant changes in allometric slopes between lakes were observed in other traits (Fig. 2b, branch length: *LR* test  = 20.8, *P* = 0.001; Fig. 2c, root/shoot ratio: *LR* test  = 6.9, *P* = 0.008; Fig. 2d, leaf number: *LR* test  = 84.6, *P* = 0.001). Branch length and leaf number were strongly positively dependent on plant size, showing an “increasing return” with increased plant size (Table 3, α>1, *P*<0.001). The two traits tended to increase more rapidly with increased plant size in Erhai Lake than that in Niushanhu Lake, as indicated by the steeper slopes. Root/shoot ratio was negatively correlated with plant size.

**Table 3:** Summary of SMA regression parameters (*n*, *p*, *r* ^2^, α, β, 95%CI, respectively) for allometric relationships between plant functional traits (shoot height, root/shoot ratio, branch length and leaf number) and plant size of the macrophyte species *Potamogeton maackianus* at two lakes (Erhai Lake and Niushanhu Lake).

Lake	*n*	*r* ^2^	*p*	α	LowCIα	UppCIα	β	LowCIβ	UppCIβ
Shoot height (cm)									
Erhai Lake	522	0.30	***	0.82	0.76	0.88	2.69	2.65	2.73
Niushanhu Lake	159	0.39	***	0.76	0.68	0.87	2.45	2.38	2.52
Root/shoot ratio (g g^−1^)									
Erhai Lake	522	0.23	***	−1.29	−1.39	−1.20	−1.86	−1.92	−1.79
Niushanhu Lake	159	0.33	***	−1.06	−1.20	−0.93	−1.69	−1.79	−1.59
Branch length (cm)									
Erhai Lake	522	0.47	***	2.22	2.09	2.37	2.95	2.85	3.04
Niushanhu Lake	159	0.66	***	1.71	1.56	1.88	2.82	2.71	2.94
Leaf number (N plant^−1^)									
Erhai Lake	522	0.48	***	1.90	1.78	2.02	2.72	2.64	2.80
Niushanhu Lake	159	0.78	***	1.18	1.10	1.27	2.66	2.60	2.72

*n*, samples number; *r* ^2^, correlation coefficient; *p*-values refer to correlation analyses following standardized major axes (SMA) procedures (see the Material and Methods section); α, slope; CIα, 95% CIs of the slope; β, intercept; CIβ, 95% CIs of the intercept;

*** , *p*<0.001.

**Figure 3:**
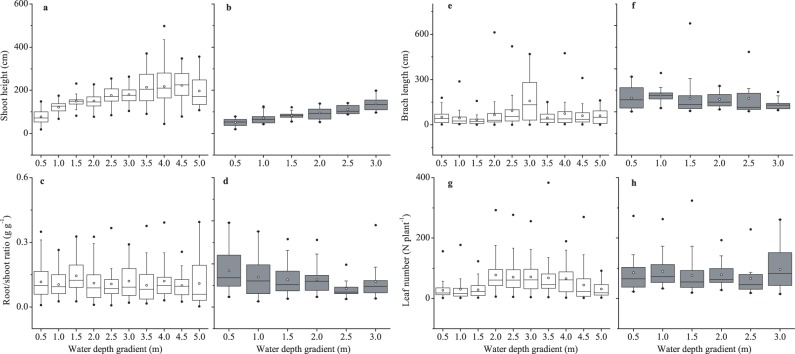
Box-plots of the four traits along water depth gradient in two lakes. (a, b) shoot height; (c, d) root/shoot ratio; (e, f) total branch length; (g, h) total leaf number. Open box indicates the data in Erhai and grey box indicates the data in Niushanhu. The median values are represented by the empty cycle, quartiles (25 and 75% percentiles) by boxes with error bars. Extreme data values are plotted with individual markers.

Except for the branch length, these traits did not exhibit significantly changes in their allometric trajectories (slope) at transect scale (all *P*>0.05), despite that they were strongly dependent on plant size (Table 4). At depth stratus scale, however, the influences of water depth gradients on the allometric trajectories of these traits were largely dependent on specific lakes (Fig. 3). In Erhai Lake, there were significant shifts in the allometric slopes between these traits and plant size along water depth gradients (shoot height: *LR* test  = 90.5, *P*<0.001; branch length: *LR* test  = 17.1, *P* = 0.001; root/shoot ratio: *LR* test  = 38.3, *P*<0.001; leaf number: *LR* test  = 26.9, *P* = 0.004). In Niushanhu Lake, in contrast, there were no significant shifts in the allometric slopes between these traits and plant size along water depth gradients (shoot height: *LR* test  = 8.02, *P* = 0.17; root/shoot ratio: *LR* test  = 9.8, *P* = 0.07; leaf number: *LR* test  = 9.9, *P* = 0.08), with the exceptions of branch length (*LR* test  = 15.7, *P* = 0.013). With increasing water depths, plants tended to increase shoot height and branch length, whereas showed no distinct trends for other two traits despite of the significant shifts in their allometric slopes.

**Table 4:** Summary of SMA regression parameters (*n*, *p*, *r* ^2^, α, β, 95%CI, respectively) for allometric relationships between plant functional traits (shoot height, root/shoot ratio, branch length and leaf number) and plant size of the macrophyte species *Potamogeton maackianus* across transects of two lakes: e1, e2, e3 in Erhai Lake and n1, n2, n3, n4 in Niushanhu Lake.

Transect	*n*	*r^2^*	*p*	α	LowCIα	UppCIα	β	LowCIβ	UppCIβ	
Shoot height (cm)										
e1	191	0.44	***	0.76	0.68	0.84	2.65	2.59	2.70	
e2	164	0.28	***	0.74	0.65	0.84	2.66	2.59	2.74	
e3	167	0.17	***	1.01	0.88	1.16	2.79	2.69	2.88	
n1	38	0.56	***	0.94	0.75	1.17	2.55	2.40	2.71	
n2	42	0.12	*	0.74	0.55	0.99	2.46	2.32	2.61	
n3	48	0.58	***	0.55	0.45	0.67	2.28	2.19	2.36	
n4	31	0.31	**	0.72	0.53	0.98	2.40	2.23	2.58	
Root/shoot ratio (g g^−1^)										
e1	191	0.19	***	−1.28	−1.46	−1.13	−1.78	−1.89	−1.68	
e2	164	0.21	***	−1.25	−1.44	−1.09	−1.83	−1.97	−1.70	
e3	167	0.20	***	−1.35	−1.55	−1.18	−1.96	−2.09	−1.84	
n1	38	0.34	***	−1.18	−1.55	−0.90	−1.77	−2.02	−1.53	
n2	42	0.35	***	−0.98	−1.26	−0.76	−1.66	−1.83	−1.50	
n3	48	0.25	***	−0.98	−1.26	−0.76	−1.65	−1.85	−1.45	
n4	31	0.28	**	−1.05	−1.44	−0.76	−1.65	−1.92	−1.39	
Branch length (cm)										
e1	191	0.55	***	2.35	2.13	2.58	2.98	2.84	3.12	
e2	164	0.44	***	2.13	1.89	2.39	2.99	2.80	3.18	
e3	167	0.39	***	2.37	2.10	2.67	2.96	2.77	3.15	
n1	38	0.77	***	1.84	1.56	2.16	3.00	2.78	3.22	
n2	42	0.75	***	1.90	1.62	2.23	2.97	2.77	3.16	
n3	48	0.73	***	1.70	1.46	1.98	2.88	2.67	3.08	
n4	31	0.64	***	1.14	0.91	1.43	2.19	1.99	2.39	
Leaf number (N plant^−1^)										
e1	191	0.52	***	1.72	1.56	1.90	2.65	2.55	2.76	
e2	164	0.38	***	1.80	1.59	2.03	2.60	2.43	2.77	
e3	167	0.40	***	2.05	1.82	2.31	2.81	2.65	2.97	
n1	38	0.85	***	1.20	1.05	1.36	2.69	2.57	2.81	
n2	42	0.58	***	1.27	1.03	1.56	2.68	2.51	2.85	
n3	48	0.81	***	1.15	1.01	1.30	2.65	2.53	2.77	
n4	31	0.88	***	1.20	1.05	1.36	2.66	2.54	2.78	

*n*, samples number; *r* ^2^, correlation coefficient; *p*-values refer to correlation analyses following standardized major axes (SMA) procedures (see the Material and Methods section); α, slope; CIα, 95% CIs of the slope; β, intercept; CIβ, 95% CIs of the intercept;

* : *p*<0.05; **: *p*<0.01; ***: *p*<0.001.

## Discussion

In this study, a widely distributed macrophyte species was used to examine the spatial structure of intraspecific trait variations and disentangle the effects of environment and ontogeny on the trait variability in each of five ecological scales. Overall, the present results lead to three important points: (1) Large amount of intraspecific variability were observed in measured traits, but the spatial structure of trait variance was largely dependent on specific scale. Shoot height might be the most important trait as response to environmental factors in lake and depth stratus scales. (2) The measured traits showed non-negligible ontogenetic variations across the five ecological scales, implying ontogenetic drifts may affect the overall traits distribution. (3) There were significant shifts in the allometric trajectories (slopes or intercepts) of measured traits at three ecological scales (i.e., lake, belt, depth stratus).

The highly intraspecific trait variability observed in this macrophyte species, especially for branch length (CV  = 121%) and leaf number (CV  = 91%), were higher than that in some terrestrial species. For example, Albert *et al.* [14] demonstrated that the intraspecific trait variability ranged from 19–49% for shoot height, 8–25% for LDMC (leaf dry mass content) and 9–29% for LNC (leaf nitrogen content) across sixteen terrestrial species; Fajardo & Piper [13] found that the intraspecific trait variability was 21% for LMA (leaf mass per unit leaf area) and 10% for WD (wood density) in a widespread tree species, *Nothofagus pumilio*. This result was impressive but probably reasonable. First, macrophyte species experience reduced gravitational force as a result of the buoyant nature of water, their morphologies and mechanical architecture are not constrained by fundamental mechanical adaptations required for self-supporting growth forms [67], [68] and are thus characterized by a great plasticity in morphological traits [69]. Second, previous studies have demonstrated that *P. maackianus* exhibited highly variations on phenotypic traits as responses to environmental gradients (i.e., water depth, light, nutrient) [41], [43], [44], [45]. Third, Li *et al.* [42] indicated that this species showed a relatively high level of genetic diversity in seven lakes of the middle reaches of the Yangtze River, which may provide genetic bases for the highly intraspecific trait variability.

Notably, many submersed macrophytes are clonal plants that mainly reproduced vegetatively during growth season [70]. *P. maackianus* generates ramets by tilling in the beginning of growth season, and all new ramets grow from the internodes of overwinter shoots. Therefore, by our sampling method, the total trait variances, especially the variations among individuals within quadrat, should be larger than using intact genet as an individual, and the variations among ramets should be underestimated in some extent due to smallest tilling shoots might be damaged by sampling dislodging. Moreover, the differences in the development stage of individuals (ramets) within a genet or among genets would be an important source of intraspecific trait variations, which was not involved in our study due to the extreme difficulty to quantify the size or boundary of a genet for this species under water.

Variance partitioning of traits demonstrated that the spatial structure of total variance was largely dependent on scale, suggesting that the environmental gradients in different scale may have an important role in shaping the trait distribution across ecological scales. According the environmental filtering framework [32], we can decompose the five ecological scales into external (lake, transect and depth stratus scales) and internal (quadrat and individual scales) filtering processes. The present study demonstrated that shoot height and branch length were greatly affected by the external filtering processes, while root/shoot ratio and leaf number by the internal filtering processes. For example, the higher individuals were sorted into the more turbid (lake scale) and deeper (depth stratus scale) water, leading to the greater frequency of higher individuals and more skewed distribution along environmental gradients. These results were consistent with recent findings of significant nonrandom assembly of individuals within single species (*Solidago canadensis*, *Bromus inermis*, and *Poa pratensis*) in old-field plant communities [71], providing an further evidence on the hypothesis that environmental filters can also sort individuals with traits values close to optimal trait values within species.

Lake and depth stratus scales accounted for the most variations of shoot height, which was more responsive to environmental factors in lake scale (productivity) than that in depth stratus (water depth gradient) scale. This species showed a higher shoot height in Erhai Lake (higher productivity) and deeper water, which is in consistent with the studies on other macrophyte species, such as *Potamogeton obtusifolius* [57], *Nasturtium officinale* [72], *Ranunculus peltatus* [73], *Myriophyllum spicatum* [74], *Potamogeton praelongus*, *Potamogeton robbinsii* and *Vallisneria americana* [58], in responses to low light and water depth in a specific ecological scale. The increased shoot height with increased water turbidity and water depth was considered as an common light harvesting strategy for both macrophyte species [55], [74] and terrestrial species [75], [76]. This may suggest that the higher plant status may be more important than the greater photosynthetic area/rates for enhancing light harvesting under shade conditions [75]. However, the higher canopy observed in population of Erhai Lake may not always mean that they have greater competitive ability (light harvesting) than population of Niushanhu Lake, which was probably just the results of adaptive responses to local environmental variation [77].

The largest amount of variation of root/shoot ratio and leaf number was explained by individual scale, but very little by quadrat scale. This indicates that quadrats have not involved the micro-habitat heterogeneity or the clonal plasticity as we assumed [78], [79], [80]. The notable high trait variations among individuals within quadrats were also found in other measured traits (LDMC and LNC) of terrestrial species [14], which may be mainly resulted from density-dependent processes. Actually, this species showed a remarkably high population density at less than 4 m depth, with 5000–12000 shoots m^−2^ in Erhai Lake as our field observations, leading to dramatic interactions among individuals within a clone or population. This result suggested that the strong effects of internal filtering processes drove the life history differentiation at a minor ecological scale, particularly for individual function specialization. For example, individuals with higher root/shoot ratio showed a greater ability to uptake nutrients from sediments [80], [81] while others with more leaf number mainly worked as light interception [80], [81]. This defect of highly individual specialization may be compensated by the clonal integration of this stoloniferous clonal species within quadrat (0.25 m^2^) [80], [81]. In this point, the facilitation rather than competition probably may exist within population, providing an alterable explanation of extremely high density and biomass of this species.

Ontogeny accounted for 20–33% of the total traits variability. Remarkably, ontogeny affected almost all measured traits across five ecological scales, with non-negligible ontogenetic variance in each scale. A recent study on the intraspecific trait variation in a widespread tree species indicated that a relative lower (for WD) or even not any (for LMA) ontogenetic variations accounted for the total traits variability [13]. There were some possible reasons for the significant different results. On the one hand, Fajardo & Piper [13] calculated ontogenetic variances by examining traits as a function of plant age rather than plant size (our study) and the distinct basis of age or size may contribute to those differences. Age-dependent ontogeny can explain most of traits variation along a real time processes (i.e., herbivory, competition, community structure and natural selection) [37]. However, size-dependent ontogeny may incorporate more comprehensive traits variation along environment gradients, because the allometric relationship between trait and plant size can reflect not only the trait responses to varying resources level but also the trait variations along the ontogenetic trajectory (different growth rates) [33], [37]. On the other hand, Fajardo & Piper [13] defined ontogeny as an independent ecological scale, while the present study nested ontogeny within each ecological scale. Actually, ontogenetic drifts may occur across spatial (from local to global) and organismal (form individual to community) scales, resulting in the changes in the traits distribution or variability across scales. Furthermore, in a laboratory experiment (single scale), Xie *et al.* [82] indicated that ontogeny accounted for a relatively low variations of root (30%) and leaf (36%) and high variations of stem (77%) for a terrestrial species (*Gossypium herbaceum* L.) under two contrasting soil texture. The relatively higher ontogenetic trait variation was largely due to the less complex environmental conditions in laboratory than that in field. Overall, our results suggested that ontogenetic variations accounted for the difference in trait responses not only among individuals within population but also among populations, and not only at local but also at regional scale.

This species showed significant shifts in allometric trajectories (slope or intercept) of measured traits in responses to external filters (lake and depth stratus). According to the allometric plasticity theory [33], the adjustments in measured traits were not only plasticity in the developmental rates but also the allometric trajectories, and thus adaptive responses to environmental factors in those two ecological scales. There are growing evidences that plant species can adjust the allometric relationships between phenotypic traits (i.e., organ mass, morphological traits and reproductive allocation) and plant size in responses to varying resource level (i.e. light, nutrient, water and water depth), although these results were generally based on an single scale [38], [39], [82], [83], [84], [85], [86]. Two recent papers have reviewed the reproductive allometry within population of terrestrial species [87], [88], and they emphasized that both environmental and ontogenetic cues have an important role in shaping the range of reproductive allometry in plant population. Interestingly, although they inferred the reproductive allometry from distinct facets, both studies indicated an analogous boundary model for the population-wide relationships between reproductive and vegetative size. This boundary model might be either linear or nonlinear, and produced by many subsets of reproductive allometry within subpopulation. In the present study, the allometric trajectories of the measured traits always showed the same direction for each measured traits regardless of scales (i.e., the allometric slopes were always positive for shoot height and negative for root/shoot ratio, Table 3 and Table 4), implying that there were probably a potential boundary for the population-wide relationships between measured traits and plant size. Furthermore, the relative contributions of ontogeny and environment to the total traits variability in different ecological scales might have a great influence on the shape of the ultimate boundary. These results would extend our understandings of the plasticity in allometric strategy within population at multi-scale.

In conclusion, understanding the source of trait variability is important in predictions of plant responses to environmental gradients. The present study quantified the spatial structure of total variability for the four important functional traits and disentangled the effects of environment and ontogeny on these traits variability across five ecological scales. This species showed a notably higher intraspecific variation in the measured traits. These traits did differ in the spatial structure, depending on environmental factors of different scales. Shoot height and branch length were most responsive to external filtering processes (lake, transect and depth stratus), while root/shoot ratio and leaf number to internal filtering processes (quadrat and individual). There were significant shifts in allometric trajectories (slope or intercept) of the four measured traits at lake and depth stratus scale, whereas the mechanisms underlying the plastic allometric strategy within population needed further multi-scale studies under clearer theoretical framework. Our results highlight that environmental filtering processes can sort individuals within species with traits values adaptive to environmental changes and ontogenetic variation of functional traits was non-negligible across the five ecological scales.
